# A CPS-Based Architecture for Mobile Robotics: Design, Integration, and Localisation Experiments

**DOI:** 10.3390/s25185715

**Published:** 2025-09-12

**Authors:** Dominika Líšková, Anna Jadlovská, Filip Pazdič

**Affiliations:** 1 Department of Cybernetics and Artificial Intelligence, Faculty of Electrical Engineering and Informatics, Technical University of Košice, Letná 9, 042 00 Košice, Slovakia; 2 School of Physics and Astronomy, The University of Birmingham, Edgbaston, Birmingham B15 2TT, UK; f.pazdic@bham.ac.uk

**Keywords:** cyber–physical systems, mobile robotics, distributed control systems, ROS2, SLAM, embedded systems

## Abstract

This paper presents the design and implementation of a mobile robotic platform modelled as a layered Cyber–Physical System (CPS). Inspired by architectures commonly used in industrial Distributed Control Systems (DCSs) and large-scale scientific infrastructures, the proposed system incorporates modular hardware, distributed embedded control, and multi-level coordination. The robotic platform, named *MapBot*, is structured according to a five-layer CPS model encompassing component, control, coordination, supervisory, and management layers. This structure facilitates modular development, system scalability, and integration of advanced features such as a digital twin. The platform is implemented using embedded computing elements, diverse sensors, and communication protocols including Ethernet and I2C. The system operates within the ROS2 framework, supporting flexible task distribution across processing nodes. As a use case, two localization techniques—Adaptive Monte Carlo Localization (AMCL) and pose graph SLAM—are deployed and evaluated, highlighting the performance trade-offs in map quality, update frequency, and computational load. The results demonstrate that CPS-based design principles offer clear advantages for robotic platforms in terms of modularity, maintainability, and real-time integration. The proposed approach can be generalised for other robotic or mechatronic systems requiring structured, layered control and embedded intelligence.

## 1. Introduction

The integration of computational elements with physical systems has led to the emergence of Cyber–Physical Systems (CPSs), a multidisciplinary field at the intersection of embedded systems, control theory, networking, and physical processes. CPSs enable real-time interaction between the digital and physical worlds, with applications spanning from industrial automation and smart grids to autonomous vehicles and medical systems [1,2]. Foundational contributions [1,3,4] established the theoretical foundations of CPSs, while more recent surveys have emphasised their evolving role in embedded and robotic systems. A key characteristic of CPSs is their layered architecture, which provides modularity, scalability, and fault tolerance. Modern CPSs often draw on established models such as Distributed Control Systems (DCSs) and Networked Control Systems (NCSs), emphasising decentralisation and interoperability [5,6].

In parallel, mobile robotics has advanced significantly, driven by progress in embedded computing, sensor technologies, and autonomous navigation algorithms. Modern mobile robots are expected to operate autonomously in complex and dynamic environments, performing tasks such as mapping, localisation, path planning, and interaction with humans or other machines. To meet these demands, robotic platforms increasingly adopt CPS principles—modular hardware, real-time software orchestration, and multilevel control—to ensure flexibility, reliability, and ease of development [7]. Recent works have also demonstrated the integration of CPS concepts with robotic middleware such as ROS2, enabling real-time communication and distributed decision-making in multi-robot scenarios [8,9].

The modelling and application of CPSs in mobile robotics is a growing area of interest, where the system architecture must account not only for the physical structure and embedded logic of the robot but also for integration with the sensing, data fusion, decision-making, and user interaction layers. Platforms that support this kind of layered design benefit from better reusability, easier diagnostics, and improved adaptability to new tasks or environments.

This paper builds on research conducted at the Center of Modern Control Techniques and Industrial Informatics (CMCT&II), at the Department of Cybernetics and Artificial Intelligence (DCAI) at the Faculty of Electrical Engineering and Informatics, Technical University of Košice. Within this context, several mobile robotic and mechatronic systems have been developed, focusing on the modelling, identification, simulation, and control of cyber–physical systems using a DCS architecture [5,10].

Within the field of mobile robotics, CMCT&II developed the mobile robots named *ModBot and MiroSot*. These robots can be analysed and classified into layers of the DCS structure. Beyond the implementation of a single mobile robot in a DCS structure, applications such as robotic football demonstrate the practical use of DCS modelling, simulation, and control methods. Robotic football has physical subsystems, such as sensors and actuators, computational subsystems—supervisory computers or microcontrollers, and a communication network connecting these subsystems [5,11,12].

In [13], the authors implemented the model of a single invented pendulum as a CPS, while using a hybrid control approach. The decomposition of the model is described based on the DCS infrastructure at CMCT&II. The same DCS infrastructure is used in [14,15] for design, mathematical modelling combined with experimental identification, and control of two different models: the aerodynamic levitation model and the magnetic levitation model.

Another example of DCS architecture can be seen within the detector control system (ALICE-DCS) of the ALICE—*A Large Ion Collider Experiment* at the Large Hadron Collider (LHC) at the European Organisation for Nuclear Research (CERN). ALICE-DCS ensures stable and safe operation of the detectors, while simultaneously controlling, monitoring, and acquiring data from the detector’s electronic system [16,17]. Within the framework of a research project entitled *ALICE Experiment at the LHC at CERN: Study of Strongly Interacting Matter in Extreme Conditions (ALICE-TUKE)*, members of CMCT&II collaborated with ALICE-DCS on the following research topics. To address increasing real-time data demands and reduce the load on the ALICE-DCS SCADA system, the ALICE Low-Level Front-End Device (ALFRED) software layer was implemented. It preprocesses data and abstracts detector electronics through a distributed architecture. At its core, the Front-End Device (FRED) framework was developed to translate low-level hardware communication into a unified DIM protocol, allowing efficient interaction with SCADA/HMI systems [10,18].

Although these implementations illustrate the flexibility and robustness of DCS-based CPSs in various domains, including mobile robotics, it is important to situate them within the broader research landscape. Recent studies have explored cyber–physical architectures in the context of mobile robotics, focussing on modularity, real-time control, and distributed decision-making [8,19]. Platforms such as those proposed in [20,21] have demonstrated the effectiveness of advanced localisation techniques such as pose graph SLAM within CPS frameworks. Other works have focused on scalability and integration in multi-robot or industrial contexts, leveraging ROS2 and digital twins to enhance coordination and fault tolerance [9,22]. Nevertheless, many of these approaches remain limited in scope, either abstracting away the physical hardware implementation or lacking a unified model that tightly integrates CPS principles with embedded control, sensing, and autonomy.

The main contribution of this paper lies in the design and realisation of a modular mobile robotic platform, named *MapBot*, designed as a layered cyber–physical system. Unlike conventional robotic implementations, in this paper we aim to integrate a DCS architecture to manage sensing, decision-making, and actuation in a decentralised and scalable manner. The paper contributes (i) a modified architectural model tailored for CPS-based robotic systems, (ii) a mapping of this model onto physical and embedded components, and (iii) an evaluation of the platform through the implementation and comparison of two localisation approaches: Adaptive Monte Carlo Localisation (AMCL) and pose graph SLAM. These contributions demonstrate how CPS principles can enhance the flexibility, maintainability, and extensibility of autonomous robotic systems.

This paper is structured as follows. The first part presents the fundamental concept of CPSs. The following part describes the infrastructures of CPSs, that are to be considered in scenarios to illustrate the modelling and development approach. The last part of the paper is devoted to the step-by-step description of the proposed approach for the design and implementation of a mobile robot within the CPS concept.

## 2. Cyber–Physical Systems—Fundamental Concept

**Figure 1:**
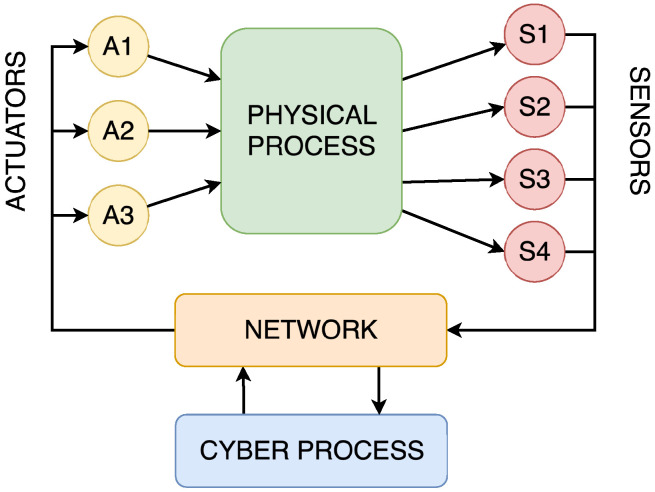
Schematic representation of the concept of CPSs.

Cyber–physical systems are frequently used to illustrate the convergence of computation, control, and communication, thereby seamlessly integrating the physical and digital worlds. CPSs can be represented as a large number of interconnected nodes, highlighting the importance of networks as they represent an essential part of the CPS. These networks ensure communication between computational processes, sensors, and actuators, leading to the interaction of computational and physical processes. To make these interactions safe, secure, and in real-time, embedded systems became an integral part of the CPS [2]. The conceptual diagram of the structure of the CPS can be seen in Figure 1.

Embedded Systems (ESs) are computer systems with specialised functionalities, frequently integrated into larger systems such as CPSs. ESs are mostly designed as small devices with the complete hardware equipment necessary to function properly and independently. ESs can also be interconnected by extensive wired or wireless networks, enabling close cooperation between individual subsystems [23,24].

Incorporating ESs within the CPS facilitates the placement of computational processes in proximity to the physical processes, thereby enabling effective real-time performance. This concept enables local-managed control of physical processes while simultaneously facilitating coordination between subsystems by the supervisory system [3,25].

**Figure 2:**
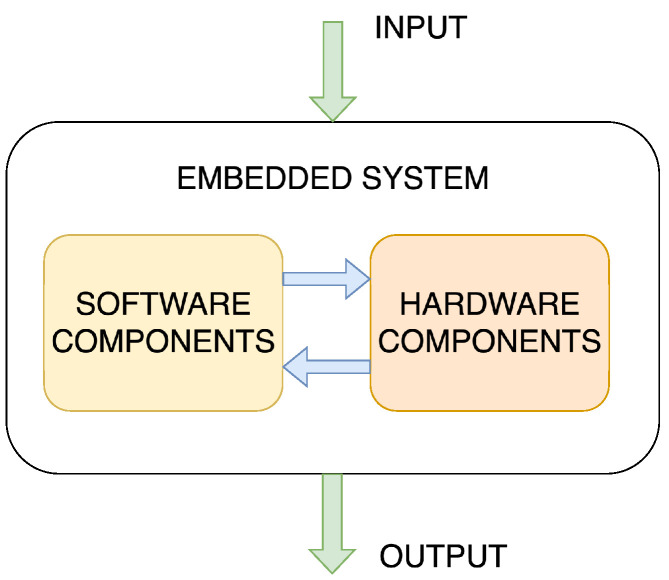
Schematic representation of the concept of ESs.

Embedded systems, regardless of their function, have a broad structure that reveals two major components, including hardware components, such as a Central Processing Unit (CPU), typically in the form of a microcontroller, and software programmes, usually included as firmware, that provide functionality to the hardware. Figure 2 illustrates this general structure, highlighting the relationship between the software and hardware component. ES inputs typically include process variables and parameters from sensors and I/O ports. These inputs are processed and used to control system actuators or provide information to users or other subsystems, representing the output of the ES [23,26].

**Figure 3:**
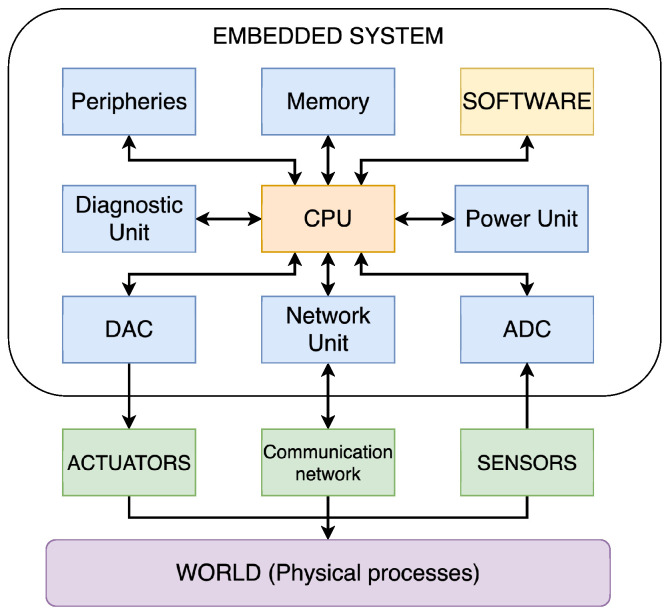
Schematic representation of the components of an ES.

From a general perspective, the hardware components of ESs include all the electronics required for the system to execute its intended function. Consequently, the specific structure of a particular system can vary significantly from another, depending on the application it serves. Despite these differences, the main components of an ES are as follows: CPU, memory, power, diagnostic, network units and peripheries, and Analogue-to-Digital (ADC) and Digital-to-Analogue (DAC) converters [26], as shown in Figure 3.

Within the CPS, the ES represents the computational part of the system. Thus, the hardware of the ES needs to be compatible with the conversion between the continuous dynamics of the physical process and the discrete dynamics of the cyber process. To ensure this compatibility, the ADC and DAC are used.

CPSs exhibit distinct characteristics that distinguish them from conventional computing and control systems. These include network and resource limitations, scalability, connectivity, adaptive reconfiguration, high level of automation, and reliability. In essence, CPSs integrate diverse components from information and communication technologies, embedded systems, networked control systems, and the Internet of Things. These characteristics must be considered when analysing, modelling, and controlling CPSs [10,27].

## 3. Analysis/Review of CPS Architectures

In terms of architecture, CPSs can employ a diverse range of structures to perform their operations. CPSs represent a widely used technology of Industry 4.0; therefore, different applications and architectures were proposed. These architectures must adhere to two fundamental principles. Firstly, they must fulfil the requirements of the system, ensuring aspects such as safety, reliability, adaptability, and real-time responsiveness. Secondly, the application goals determine the structure of the whole CPS [28].

Among the most widely used architectures of CPSs are, for example, layered architectures, cloud-based architectures, Service-Orientated Architectures (SOAs) that facilitate modular and flexible system integration, and event-driven architectures that respond dynamically to real-time stimuli [5,29,30,31]. To illustrate the practical deployment of these architectural styles in various domains, Table 1 presents a selection of representative CPS architectures, their associated use-cases, and the corresponding functional layering. This compilation highlights the diversity and adaptability of architectural patterns tailored to meet the specific demands of domains such as industrial automation, smart grids, healthcare, and transportation systems.

The comparative overview in Table 1 shows that layered architectures are a unifying feature of CPS design, although their implementations diverge between domains. Industrial automation follows the five-layer ISA-95 hierarchy, ensuring coordination from devices to enterprise management [32], while smart grids adopt four-layer decentralised designs with sensing, local, and grid controllers linked to the cloud [33]. Scientific instrumentation, such as the ALICE detector at CERN, also employs five layers, including SCADA, database, and management for large-scale monitoring [5]. Other domains adopt four-layer models, such as e-Health systems that use IoT-enabled CPSs with devices, gateways, cloud analytics, and clinical interfaces [34]; smart agriculture integrates sensors, edge, cloud, and decision logic [36]; and smart mobility combines sensors, vehicle control, V2X communication, and cloud coordination [37]. Smart buildings extend this four-layer structure to human-centric interaction, embedding occupants as active participants in sensing, control, and optimisation [35]. In contrast, several CPS frameworks in the literature describe only three layers: physical, network, and application [38] or environment, communication, and cyber layer [39], offering a simplified baseline abstraction. In comparison, domain-specific architectures of four and five layers reflect additional needs such as enterprise integration, SCADA management, or human-in-the-loop optimisation.

Layered architectures are a common feature in CPS domains, and these abstractions are often realised in practical systems. Distributed Control Systems (DCSs) in industrial automation provide a clear example in implementing CPS principles in real-world deployments. DCSs share key characteristics with CPSs, including distributed sensing, computation, and control, offering performance, reliability, and scalability benefits [6]. Rather than relying on a single central controller, DCSs distribute tasks throughout the system, placing control elements close to the physical processes they manage. The supervisory systems oversee these components, providing a complete view of operations. Architectures typically follow multiple functional or control levels defined in standards such as IEC 62264 (ANSI/ISA-95) [40], although the naming and numbering may vary [5,41,42].

**Figure 4:**
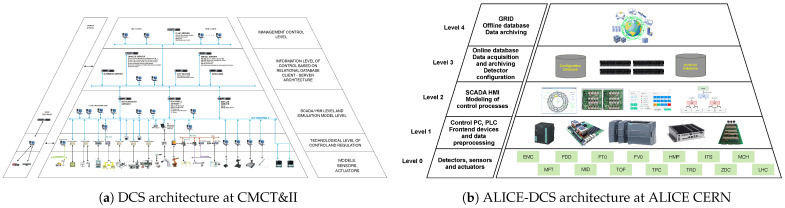
Schematic representation of the illustrative examples of the architectures.

Inspired by these principles, mobile robots can be designed as a CPS integrated into a DCS framework. An implementation at CMCT&II, DCAI, is illustrated in Figure 4a. This five-level architecture supports individual CPS subsystems at each layer, forming a complex hierarchical CPS. Physical systems, such as mechatronic devices modelled as CPSs, can be integrated at all levels [13,43].

Similar layered patterns are also found in other domains, including large-scale detector control systems [5]. An illustrative example of this can be found within the ALICE experiment at the Large Hadron Collider (LHC), CERN, which employs a five-layer Detector Control System (ALICE-DCS)—Figure 4b—comprising devices, control of sub-detectors and front-end electronics, SCADA/HMI visualisation, and information management incorporating databases for storing the data from lower-level and high-level supervisory coordination [16]. This structure mirrors the hierarchical principles seen in industrial DCS implementations, illustrating the broad applicability of layered CPS architectures across domains.

In summary, the analysis of representative CPS architectures and use cases demonstrates that layered structures are a versatile principle across domains. From industrial automation and smart grids to healthcare, smart mobility, and scientific instrumentation, hierarchical organisation supports distributed sensing and control, system-level coordination, and integration of cyber and physical components. Examples such as the DCS at CMCT&II and ALICE-DCS at CERN illustrate how these principles are implemented in practice, highlighting the adaptability of multilayered architectures to diverse operational requirements. Building on this analysis, the following sections will focus on the implementation of the multilayered CPS architecture and its application within the field of mobile robotics. 

## 4. Considered Cyber–Physical System

**Figure 5:**
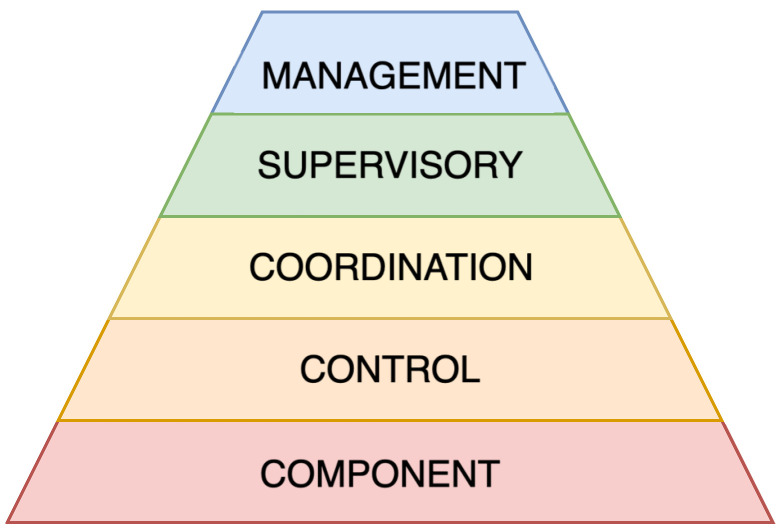
Schematic representation of modified CPS architecture.

In the literature, CPSs have been described using a variety of architectural models. The simplest approaches are based on three layers [38,39], while others extend this to four layers, often adding elements such as edge computing or cloud-based analytics [34,36]. These models provide a clear starting point but can be too abstract to capture important features such as distributed coordination, supervisory control, or human interaction. However, industrial standards such as ISA-95 define five hierarchical levels that address these needs in detail, but their terminology and focus are tailored specifically to manufacturing and DCSs. This makes them less suitable as a general framework for CPSs in other domains. To address this gap, we propose a modified five-layer architecture (Figure 5) that preserves the hierarchical clarity of ISA-95 while introducing domain-independent terminology and extending its applicability beyond industrial automation. In contrast to minimal 3–4 layer models, the architecture explicitly distinguishes between control, coordination, supervisory, and management layers, allowing for finer-grained modelling of distributed and scalable CPSs. Furthermore, the management layer incorporates the digital twin concept, enabling predictive decision-making and system optimisation. In this way, the proposed model provides a general and flexible framework, applicable to robotics, mechatronics, large-scale scientific instrumentation, and other CPS domains.

The lowest layer, *Component*, represents the hardware, such as sensors and actuators. The *Control* layer corresponds to low-level control elements, such as microcontrollers, that ensure the basic functions of the system. Above this, the *Coordination* layer represents a higher level of control responsible for more complex tasks, including information exchange between the lower and upper layers of the CPS. The subsequent *Supervisory* layer encompasses user interfaces, Human–Machine Interfaces (HMI), and Supervisory Control And Data Acquisition (SCADA) systems. At the top, the *Management* layer represents the decision-making processes, system deployment, and the implementation of the digital twin.

### 4.1. Integration of the CPS Architecture Within the Mobile Robot Platform

**Figure 6:**
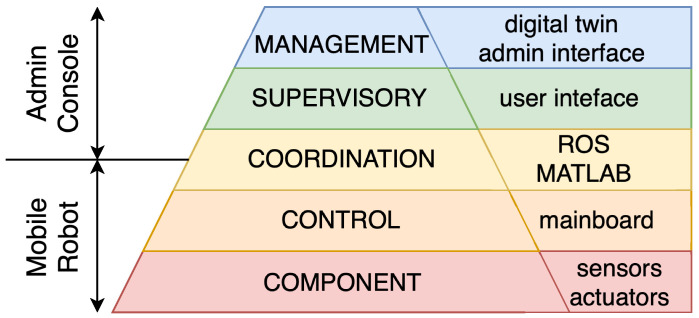
Schematic structure of integration of the CPS architecture within the mobile robot platform.

Although CPS architectures are often described in abstract terms, their practical implementation requires careful integration of hardware and software components. The CPS architecture is mapped to the physical modules and computational processes of the described mobile robot platform—Figure 6.

Based on the previously introduced CPS architectural model and the principles of embedded systems and hardware modularity, the mobile robot platform was developed as a layered cyber–physical system. The component layer consists of the physical hardware, including sensors (stereo cameras, LiDAR, IMU, ToF) and actuators (drive motors). The control layer is implemented through low-level embedded elements, such as the drive board and microcontrollers, which manage motor control and sensor interfacing. The coordination layer is realised by the mainboard and expansion modules that handle data exchange and coordination between the sensing and actuation subsystems. The supervisory layer includes the interfaces used for monitoring and interaction, namely the Steam Deck for manual control and the visualisation tools (e.g., RViz) operating on user PCs. Finally, the management layer supports high-level decision-making, system-level orchestration, and the integration of a digital twin, which provides a virtual representation of the robot and its environment for monitoring, simulation, and predictive control. Together, these layers form a coherent CPS implementation, where cyber and physical domains interact in real time through standardised interfaces and communication protocols.

The integration of the mobile robot platform into the CPS architecture establishes a structured foundation for modular development, layered communication, and scalable control. With the hardware and software components clearly mapped to architectural layers, the system is now ready for advanced functional design.

### 4.2. Design of Mobile Robot

**Figure 7:**
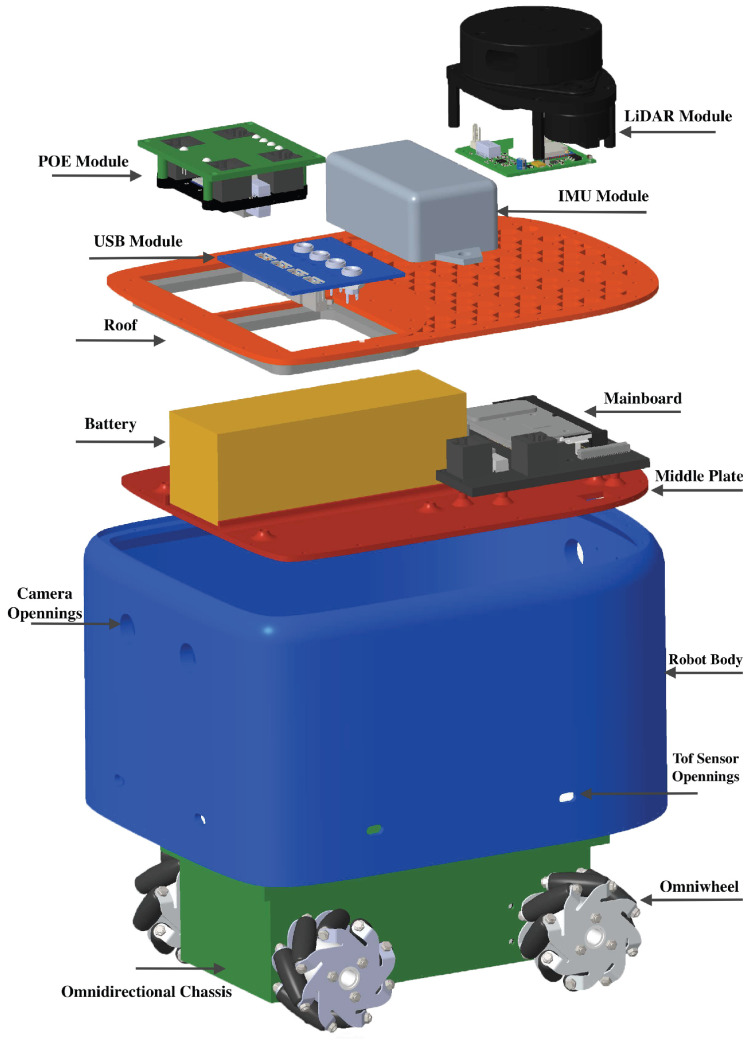
Three-dimensional rendered model of mobile robot with disassembled skeleton.

The design of the robotic platform, called *MapBot*, is modular and consists of several main components; see Figure 7. This design enables future modifications and replacements of individual components without the need to alter the entire construction. Individual modules can also be used for other applications.

The robot has a single-piece oval body with a central plate that secures the electronics and the chassis. It can hold two stereo cameras (front and rear) and nine TOF sensors around the lower perimeter (Figure 7).

The mainboard is based on the Nvidia *Jetson Nano*, enhanced with an NVMe slot for high-speed storage and a *HiLink 7628* router for network connectivity. It also includes one USB 2.0 port for hubs or modules. The robot roof is a single plate, either 3D-printed or laser-cut. The roof can be divided into a grid with threaded openings to mount the expansion modules and a connectivity section for POE or USB expansion hubs.

The chassis features four motors with worm gears and encoders in a 3D printed body. Omnidirectional wheels with rollers placed at $45^{\circ }$ are used to allow movement in all directions. The ESP32 microcontroller manages motor control via a dual H-bridge (*A4990*) and an IO expander (*PCA9555*) to connect all encoders and outputs.

*MapBot* supports various expansion modules, including a POE module that provides data and power to up to four modules via Ethernet, a LiDAR module aligned with the robot’s centre for accurate environmental mapping, and an IMU module placed at the roof centre for precise positioning beyond wheel odometry.

**Figure 8:**
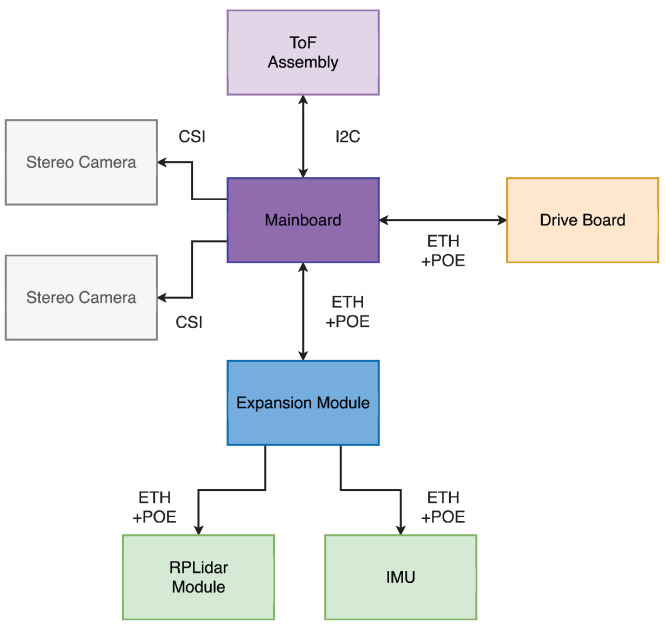
Schematic diagram of networking solutions within the robot’s modules.

The communication architecture is modular. The mainboard handles high-bandwidth data from stereo cameras (CSI) and TOF sensors (I2C), while the drive board is controlled over Ethernet with POE. Expansion modules such as LiDAR and IMU also connect via ETH+POE, serving as communication hubs for peripherals (Figure 8).

The system runs on ROS2, distributed in the Jetson Nano, a user PC, and a Steam Deck. Each executes different subsystems: low-level control and sensor drivers on the Jetson, mapping and visualisation on the PC, and manual control on the Steam Deck. This modular hierarchy ensures scalable and robust integration of perception, control, and localisation within the CPS design of the robot.

## 5. Application Library for Proposed Mobile Robot Platform

To ensure specific behaviour and validate control strategies before deployment, a comprehensive modelling and simulation framework must be developed. This framework forms the basis of a digital twin of the *MapBot* robot, providing a virtual representation that mirrors the physical system in real time. The proposed library is composed of two main parts: the first is a simulation module that replicates the robot’s kinematic and dynamic behaviour, the other implements functionality of the real model of the mobile robot and an interface that connects the simulation to the actual hardware, enabling seamless testing and validation.

### 5.1. Mathematical Modelling of Mobile Robot

The mathematical model represents a suitable form of description of a physical model, such as a mobile robot. To create a simulation model and propose a control algorithm, the mathematical model is a necessary component of this process. The mathematical model of a mobile robot can be described by kinematic and dynamic models.

#### 5.1.1. Kinematic Model of Mobile Robot

In general, the kinematic model defines the relationship between the velocities of a robot’s joints and the resulting motion of its end-effector or body. In the context of mobile robotics, wheels serve as joints and their rotational velocities determine the robot’s movement in space. Consequently, the kinematic behaviour of a mobile robot is inherently dependent on the configuration of its chassis and wheel arrangement.

**Figure 9:**
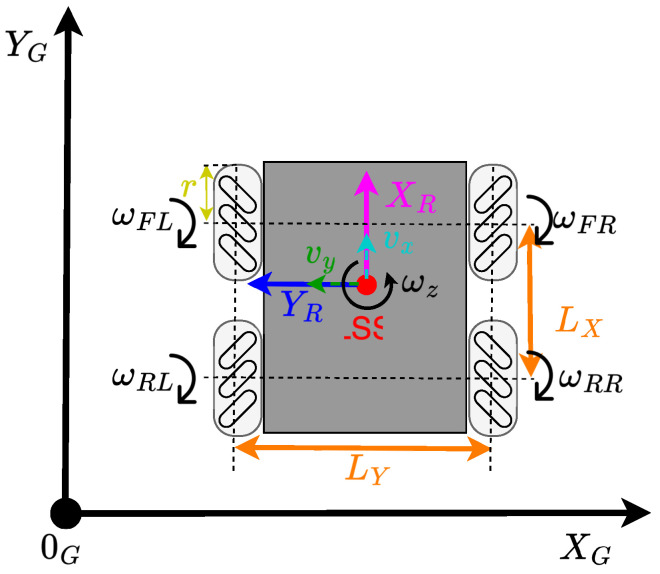
Schematic structure of the chassis with omnidirectional wheels.

The mobile robot platform used in this paper, called *MapBot*, features a four-wheeled chassis equipped with Swedish omnidirectional wheels, as illustrated in Figure 9. This wheel configuration allows for holonomic motion, which is particularly advantageous for tasks such as Simultaneous Localisation and Mapping (SLAM). To achieve omnidirectional movement, the robot uses two diagonally positioned wheels with rollers inclined at positive angles, while the remaining two wheels have rollers inclined at negative angles.

The physical quantities and parameters shown in Figure 9, essential for building the kinematic models, are summarised in Table 2 and Table 3.

Based on the configuration of the mobile platform, *MapBot* is controlled by four independent wheel velocities $\left(\omega_{FL},\omega_{FR},\omega_{RL},\omega_{RR}\right)$. The forward kinematics $f_{f}$ of the system, which describes the relationship between individual wheel speeds and the resulting linear $\left(v_{x},v_{y}\right)$ and angular $\omega_{z}$ velocities of the robot, can be expressed as:

(1) $$\left(v_{x},v_{y},\omega_{z}\right)=f_{f}\left(\omega_{FL},\omega_{FR},\omega_{RL},\omega_{RR}\right)$$

The resulting translational velocity $v_{r}$ and the orientation of the movement ρ in the robot’s local coordinate system can be calculated as:

(2) $$v_{r}=\sqrt{v_{x}^{2}+v_{y}^{2}}\;\rho =tan^{-1}\left(\frac{v_{x}}{v_{y}}\right)$$

The robot’s displacement in the global coordinate frame is then computed using:

(3) $$\begin{array}{c} \Delta X_{GSS}=v_{x}\cdot cos\left(\theta \right)-v_{y}\cdot sin\left(\theta \right)\\ \Delta Y_{GSS}=v_{x}\cdot sin\left(\theta \right)+v_{y}\cdot cos\left(\theta \right)\\ \Delta \theta_{GSS}=\omega_{z} \end{array}$$

where θ denotes the robot’s current orientation in the global reference frame.

Following the forward kinematic $f_{f}$ formulation, the inverse kinematics $f_{i}$ defines the required angular velocities of the individual wheels $\left(\omega_{FL},\omega_{FR},\omega_{RL},\omega_{RR}\right)$ based on the desired linear and angular velocities $\left(v_{x},v_{y},\omega_{z}\right)$ of the robot. This relationship can be expressed as:

(4) $$\left(\omega_{FL},\omega_{FR},\omega_{RL},\omega_{RR}\right)=f_{i}\left(v_{x},v_{y},\omega_{z}\right)$$

#### 5.1.2. Dynamic Model of Mobile Robot

While the previous section focused on the kinematic description of the robot, which captures the relationship between wheel velocities and the robot’s motion without considering inertial effects, a dynamic model is necessary to account for forces and torques involved in the robot’s motion. This is particularly important for accurate control design, simulation of physical behaviour, and interaction with real-world environments.

The dynamic equations of motion for the robot body are given by:

(5) $$M\dot{v}+Dv=F$$

where:

$v={\left[\begin{array}{ccc} v_{x} & v_{y} & \omega_{z} \end{array}\right]}^{T}$ is the velocity vector in the robot frame.
$M=\mathrm{diag}(m,m,I_{z})$ is the mass/inertia matrix.
*D* is a diagonal matrix that represents viscous friction: $D=\mathrm{diag}(d_{x},d_{y},d_{\omega })$.
F is the generalised force vector produced by the wheels: $F=J^{T}\tau$.

Motor torques $\tau ={\left[\begin{array}{cccc} \tau_{FL} & \tau_{FR} & \tau_{RL} & \tau_{RR} \end{array}\right]}^{T}$ are assigned to the chassis based on forward and inverse kinematics using the Jacobian *J*:

(6) $$J=\frac{r}{4}\left[\begin{array}{cccc} 1 & 1 & 1 & 1\\ -1 & 1 & 1 & -1\\ -\frac{1}{L_{x}+L_{y}} & \frac{1}{L_{x}+L_{y}} & -\frac{1}{L_{x}+L_{y}} & \frac{1}{L_{x}+L_{y}} \end{array}\right]$$

The DC motor dynamics for each wheel are given by:

(7) $$\tau_{i}=J_{m}{\dot{\omega }}_{i}+b\omega_{i}$$

(8) $$V_{i}=Ri_{i}+L\frac{di_{i}}{dt}+K_{e}\omega_{i},\;\tau_{i}=K_{t}i_{i}$$

where, for each motor:

$J_{m}$ is the moment of inertia.
*b* is the damping coefficient of the motor.
*R*, *L* are the motor winding resistance and inductance.
$K_{t}$, $K_{e}$ are torque and back-EMF constants.
$\omega_{i}$ is the angular velocity of the *i*-th wheel.
$V_{i}$ is the applied voltage.

The ideal conditions assumed in Equation (5) provide a dynamic model of first order, but omits some of the real-world criteria. The mobile robot is assumed to have a constant mass when designing the dynamic model. To achieve a more precise model, consideration of varying mass and inertia due to payload variation is required. In addition, certain approaches involve the consideration of the interaction between the wheels and the ground, which is constrained by a friction coefficient. The friction coefficient may change according to the surface and the slip of the wheel. Multiple techniques have been developed to deal with friction models, such as the dynamic modelling approach for wheeled robots that incorporates the estimation of friction coefficients on different floor surfaces [44], slip control of wheeled robot while climbing the step by estimating the contact angle [45], or fuzzy sliding-mode control for the task of trajectory-tracking with an autonomous differential drive robot [46].

**Figure 10:**
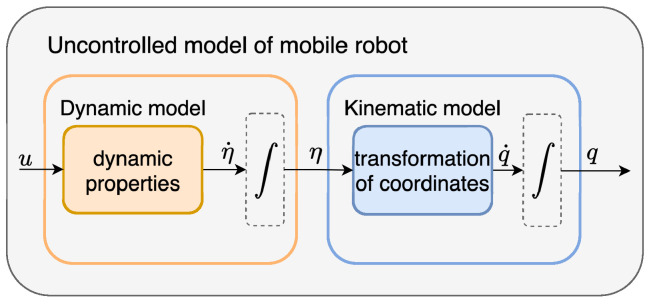
Conceptual diagram of uncontrolled model of mobile robot.

The conceptual diagram—Figure 10—illustrates the structure of an uncontrolled mobile robot model, divided into two main components: the dynamic model and the kinematic model. The input *u*, representing the motor commands (e.g., torques or voltages), affects the robot’s dynamic properties, resulting in the evolution of the dynamic state derivative $\dot{\eta }$. This signal is integrated to produce the velocity state η, which is then fed into the kinematic model. The kinematic model performs a transformation of coordinates to compute the configuration rate $\dot{q}$ in the global frame, which is subsequently integrated to obtain the robot’s final configuration *q* (e.g., position and orientation). This modular decomposition supports independent modelling and analysis of the robot’s dynamics and kinematics, simplifying control system design.

This model can be expressed as a state-space system by combining the robot and motor states. Since not all parameters of the DC motors used in the mobile robot were available, each motor was modelled based on experimentally identified characteristics.

#### 5.1.3. Experimental Identification of DC Motor

The mathematical representation of the DC motor reveals that it behaves as a second-order system, due to the interconnection between its electrical and mechanical dynamics. The DC motor model was obtained using the MATLAB (R2024b) System Identification Toolbox. The identification process involved several steps: acquisition and preprocessing measurement data from the motor, application of identification methods within the toolbox—specifically the *ARMAX—autoregressive moving-average with extra input* model structure—followed by testing and validating the resulting model to ensure its precision.

**Figure 11:**
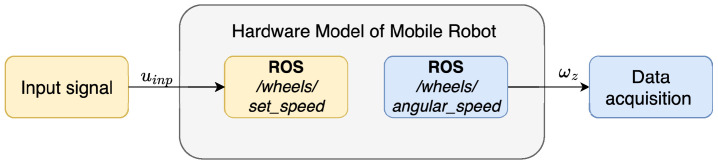
Schematic representation of the data acquisition for experimental identification.

To acquire motor data, a connection was established between Simulink and the mobile robot platform using the ROS Toolbox—Figure 11. A pseudorandom binary signal was applied as input u(t), transmitted from Simulink to the robot, while the resulting wheel speeds $\omega_{z}\left(t\right)$ were sent back to Simulink for analysis.

**Figure 12:**
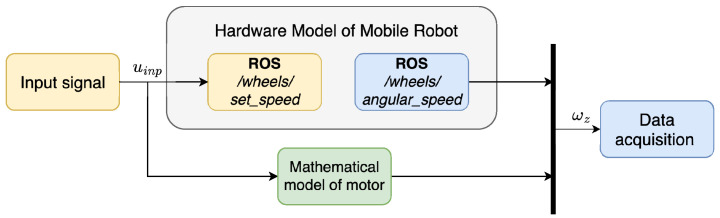
Schematic representation of the model testing and validation.

The collected data were then used to identify an ARMAX model for each motor. Subsequently, these models were tested by applying the same input signal used during data acquisition. The signal was sent simultaneously to the mathematical models implemented in Simulink and to the physical robot—Figure 12. The resulting outputs were compared to assess the accuracy of the model.

**Figure 13:**
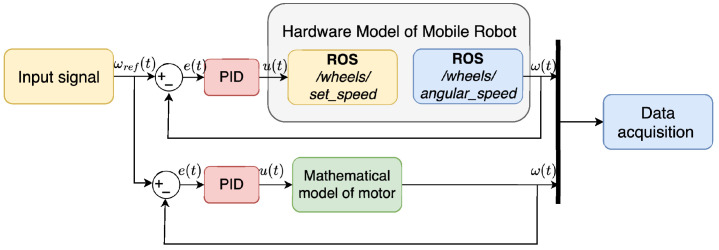
Closed-loop schema for the speed control of the motor’s speed.

Finally, model validation was performed using an open-loop scheme, the same as for the testing. Next, the closed-loop control scheme with a discrete PID controller was used—Figure 13. In this stage, the input signal was changed to a sine wave, and the responses of the motors models and the actual robot were compared to further verify model behaviour.

**Figure 14:**
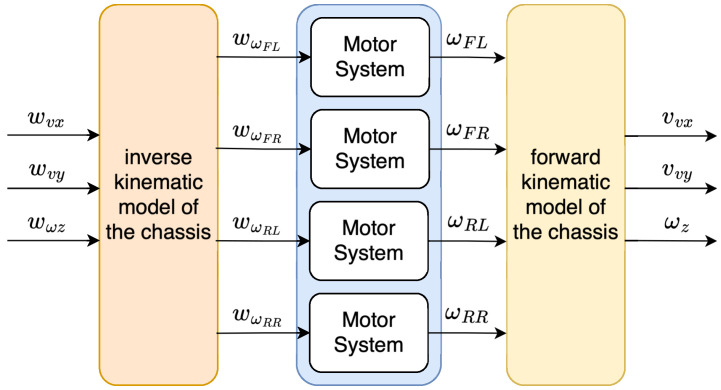
Schematic representation of the simulation model of the chassis.

Experimental identification facilitated the creation of a comprehensive simulation model of the *MapBot* platform, thus clarifying the models for each motor used on the actual robot platform. To simulate the behaviour of the robot’s chassis, the model of the chassis was implemented in a way as shown in Figure 14.

The combination of these models provides the core of the digital twin, where the virtual robot accurately reflects the motion and response of the physical robot. The experimental identification of DC motors ensures that the simulation captures realistic dynamics, allowing reliable predictions of robot behaviour before deployment.

### 5.2. Controller Design

Since the primary objective of the *MapBot* platform is the implementation of a SLAM algorithm, two preparatory tasks were conducted beforehand. The control algorithms including speed control and trajectory tracking are tested and tuned within the digital twin environment. By integrating the *MapTool* application with the simulation, the digital twin allows for real-time interaction, where control inputs can be applied to both virtual and physical robots. This approach enables the early detection of potential issues, validation of control strategies, and performance optimisation without risk to the actual hardware.

As described previously, the speed of each motor is regulated using a discrete PID controller. However, to enable coordinated motion of the entire *MapBot* platform an additional upper layer must be implemented. This layer is responsible for translating the desired motion of the robot into individual motor commands, and vice versa. Specifically, it requires converting the robot’s global velocity commands into its local frame of reference. The transformation equations used for this conversion are given by:

(9) $$\begin{array}{c} v_{x}=v_{xg}\cdot \cos\left(\theta \right)+v_{yg}\cdot \sin\left(\theta \right)\\ v_{y}=-v_{xg}\cdot \sin\left(\theta \right)+v_{yg}\cdot \cos\left(\theta \right)\\ \omega_{z}=\omega_{zg} \end{array}$$

**Figure 15:**
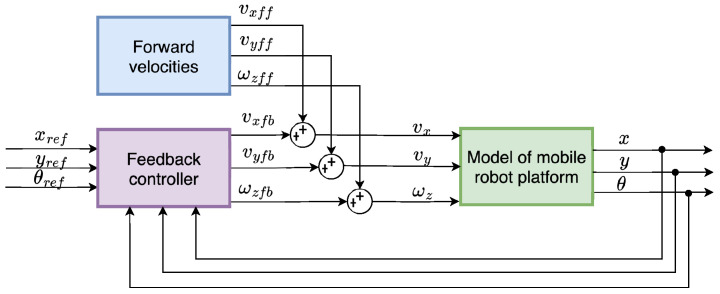
Schematic representation of the FFC for trajectory-following algorithm.

To implement the trajectory-following algorithm, Feed-Forward Control (FFC) was chosen, and its schematic representation is shown in Figure 15. The control law u(t) consists of feed-forward component $u_{ff}\left(t\right)$ and a feedback control component $u_{fb}\left(t\right)$. Thus, the velocities of the robot are defined as follows:

(10) $$\begin{array}{c} v_{x}=v_{xff}+v_{xfb}\\ v_{y}=v_{yff}+v_{yfb}\\ \omega_{z}=\omega_{zff}+\omega_{zfb} \end{array}$$

The feed-forward velocities $v_{xff},v_{yff},\omega_{zff}$ are determined from the reference trajectory. For an omnidirectional robot, these velocities can be computed in two distinct modes: either by following the trajectory while also tracking the robot’s orientation, or by following the trajectory without considering orientation tracking.

When calculating the velocity while tracking the robot’s orientation change, we assume that $v_{y}=0$ and that the robot will follow the trajectory and its orientation as if it were a differential drive. For calculating the velocity without tracking the robot’s orientation change, it is considered $\omega_{zref\theta }=0$, which means that the trajectory following is dependent only on lateral velocities $v_{xref},v_{yref}$. This mode of trajectory following is useful mainly for tasks where the robot’s orientation has to be fixed—for example, when the camera is tracking the object in motion while the robot is following the referenced trajectory. In such a case, $\theta_{ref},\omega_{zref}$ need a separate controller based on the expected behaviour.

All modules described in this section collectively form the application library for the *MapBot* robot. Together, the simulation and hardware interface constitute a digital twin, which serves as a platform to develop, test, and validate SLAM and other behaviours, as demonstrated in the case study in the following section.

## 6. Case Study

The application library developed for the *MapBot* platform serves as a unified framework for the simulation and deployment of control strategies in the real robotic system. This library enables comprehensive testing of the robot’s behaviour, evaluation of its performance in varying environmental conditions, and verification of control and navigation algorithms in a consistent and reproducible manner. In this section, the practical use of the library is demonstrated through a case study that showcases the robot’s capabilities, including speed regulation, trajectory tracking, and preparation for higher-level tasks such as SLAM.

### 6.1. Mathematical Model and Experimental Identification of DC Motors

Based on the previously introduced mathematical model and hardware setup, an experimental identification was performed to obtain accurate dynamic parameters of the DC motors used in the *MapBot* platform. Since not all required parameters were available from the data-sheets, a data-driven approach was necessary to refine the model for control and simulation purposes.

**Figure 16:**
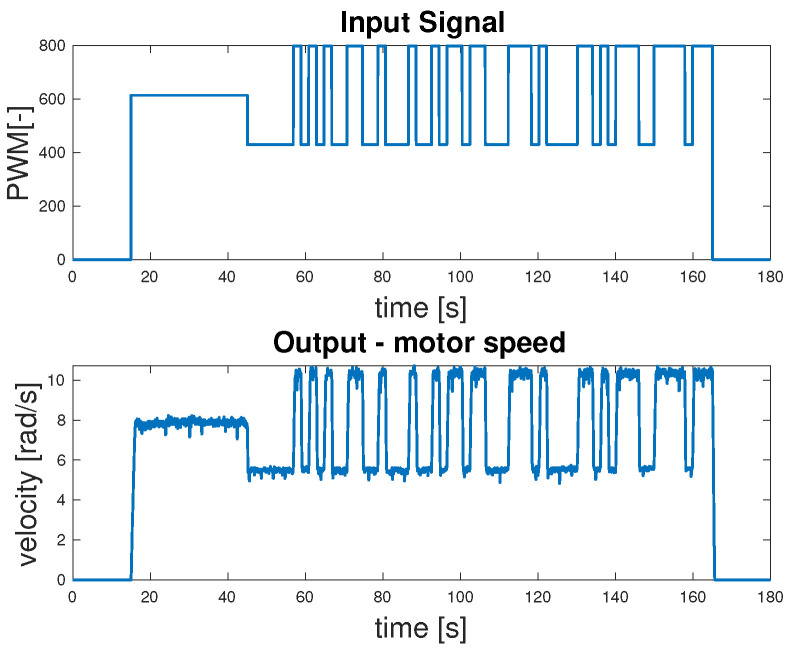
Experimental data used for ARMAX model identification.

In order to identify the ARMAX model of the DC motor, input and output data were collected based on the setup illustrated in Figure 11. The measurement resulted in the acquisition of the input signal in the form of PWM values and the corresponding output signal representing the wheel velocity, as shown in Figure 16.

**Figure 17:**
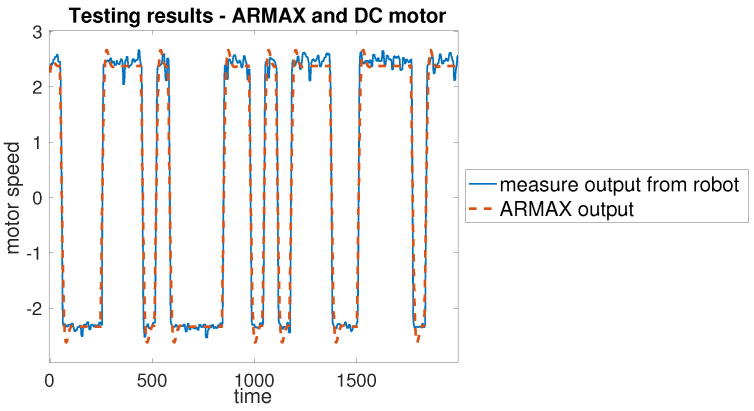
Results of the test experiment for the DC motor.

The raw data obtained directly from the robot were divided into two sets: one for model identification and another for the validation. For each motor, a dedicated subset of measurements was collected individually before combined experiments were carried out. Multiple trials were performed to ensure an accurate estimation of the ARMAX parameters. The results of all trials were compared and the most representative measurement set was selected for each motor. The best-case measurements for each motor were then used as a basis for developing the control algorithm. Figure 17 presents a representative comparison between the identified ARMAX model and the measured motor response during open-loop testing.

**Figure 18:**
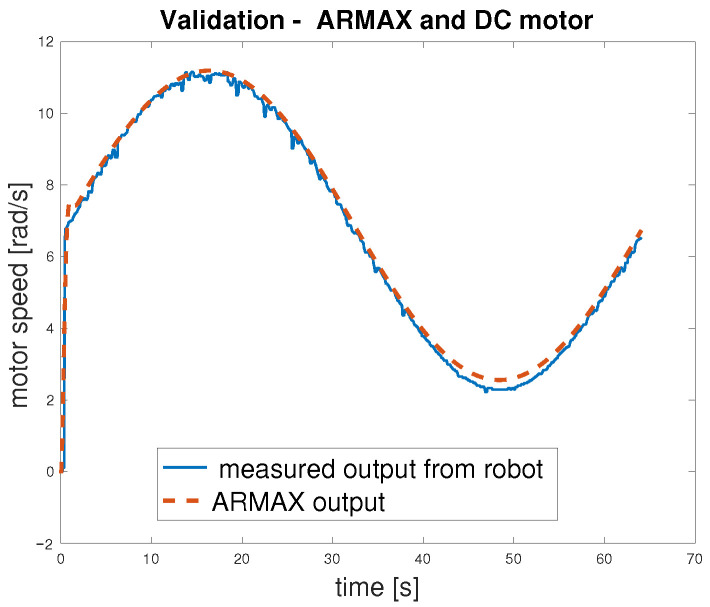
Results of the validation for the DC motor.

To verify that the motor exhibits consistent behaviour under varying operating conditions, an open-loop validation was performed within the concept shown in Figure 12. The input signal was changed from a pseudorandom binary sequence to a sine wave, which effectively covers the motor’s operational range. The validation procedure was applied as follows: the sine wave input was delivered simultaneously to both the ARMAX model and the actual DC motor within the *MapBot* platform. The results, presented in Figure 18 and summarised in Table 4, confirm that the ARMAX model adequately reproduces the real motor behaviour and is therefore suitable for the design of the subsequent control algorithm. Since very similar outcomes were observed across all four motors of the *MapBot* platform, only one representative validation example is shown.

The results in Table 4 show the error between the ARMAX model and the measured angular speed of the DC motor in open loop. Although the model captures the system dynamics reasonably well, the relatively high maximum error (3.50 rad/s) indicates that some deviations occur, particularly under transient conditions. However, the median and percentile values $\left(p_{95},p_{99}\right)$ suggest that most of the error remains within acceptable limits for control design purposes.

### 6.2. Verification of Control Algorithms

Before deploying the control algorithm on the physical platform, it is essential to verify its functionality and performance in a simulated environment. This subsection presents the verification process, demonstrating that the controller can achieve the desired robot behaviour under various operating conditions. The results provide a foundation for safe and reliable implementation in a real system.

As the ARMAX models of the DC motors were already validated, they were subsequently used to design speed controllers for each motor. A discrete PID control strategy was selected, and each motor was assigned a dedicated controller tuned according to the parameters of its corresponding ARMAX model. The behaviour of the controllers was first verified within a simulation environment. Following this, various operational scenarios were tested. Due to established ROS-based communication between the simulation and the physical *MapBot* platform, the control loop, including all computations, was executed in Simulink. Control signals were transmitted to the robot, while real-time velocity feedback was received from the motors. After confirming the correct functionality through this setup, the PID controllers were also implemented directly on the *MapBot* platform. The developed application library supports both control approaches, allowing users to select between simulation-based or on-board execution.

**Figure 19:**
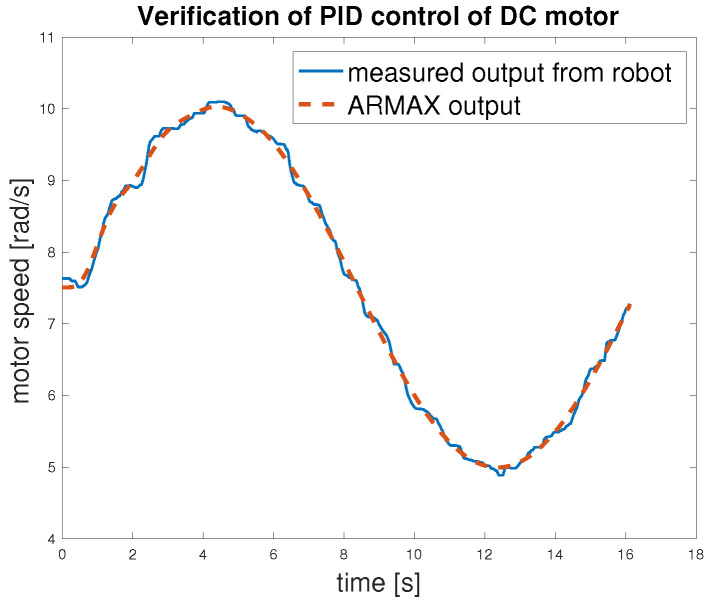
Verification of speed control of DC motor.

The closed-loop validation (Figure 19) demonstrates the performance of the proposed control algorithm. All error metrics (Table 5) are significantly lower than in the open-loop validation case, with the RMSE dropping to 0.079 rad/s and the maximum error limited to 0.22 rad/s. The small $\left(p_{95},p_{99}\right)$ values confirm that the controller ensures stable and accurate regulation, even in worst-case scenarios. This validates the effectiveness of the control strategy.

To enable the robot to follow a predefined path, a reference trajectory must first be generated. This is accomplished by defining parametric equations that describe the desired motion of the robot in time. These equations are used to compute discrete points along the trajectory, which are then interpolated to ensure a smooth and continuous motion profile suitable for control execution. The resulting set of reference points includes position and orientation data that can be sent to the *MapBot* platform as input for the motion control algorithm. This approach enables precise trajectory tracking and facilitates testing of the robot’s ability to perform navigation tasks under realistic conditions.

**Figure 20:**
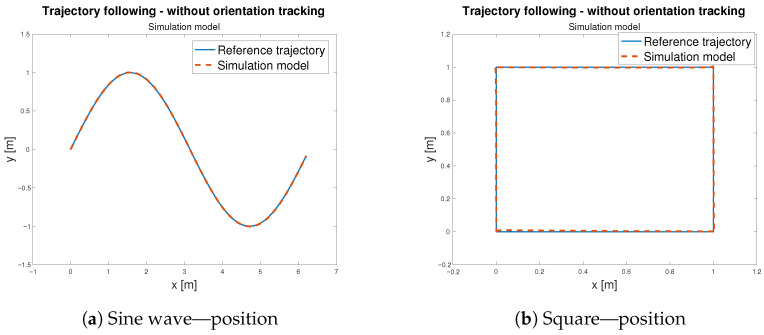
Trajectory following—simulation model validation.

To verify the functionality of the feed-forward controller defined in Equation (10), with schematic representation in Figure 15, three distinct reference trajectories, a sine wave, a square path, and a spiral path, were selected for testing. During trajectory tracking, two operational modes were considered: one in which the robot follows both position and orientation and another in which only the position is tracked Both modes were evaluated using the selected trajectories. Initially, the controller was tested in the simulation environment using the developed model—Figure 20. Subsequently, the same control logic was implemented on the physical *MapBot* platform, allowing direct comparison of the robot behaviour in simulation and in real-world conditions. This process ensured consistency and validated the effectiveness of the proposed controller.

The corresponding Euclidean tracking errors are reported in Table 6. The results confirm that the model exhibits high accuracy for both trajectories, with very small errors in the sine wave case (RMSE ≈ 2.3 mm). The square trajectory, which imposes sharper transitions and thus more demanding dynamics, naturally leads to slightly larger errors (RMSE ≈ 7.9 mm). Nevertheless, the overall error levels remain low, demonstrating that the simulation model is sufficiently accurate at reproducing the behaviour of the system for subsequent control design and validation tasks.

**Figure 21:**
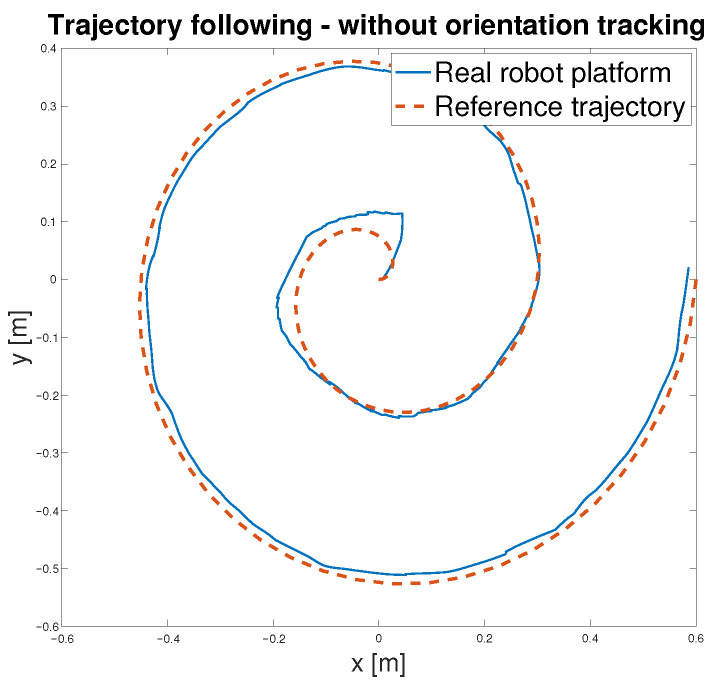
Trajectory following—*MapBot* real platform validation.

After validating the control strategy in simulation, the same algorithm was applied to the real *MapBot* platform in order to assess its trajectory tracking performance in real-world conditions (see Figure 21).

Table 7 reports the Euclidean errors between the reference and the actual motion of the *MapBot* platform. Position tracking exhibits an RMSE of approximately 2.8 cm, while the orientation error remains lower, with an RMSE of about 0.017 rad. Although larger deviations occasionally occur (maximum errors of 5.9 cm in position and 0.075 rad in orientation), the 95th and 99th percentile values indicate that the system maintains consistent accuracy during most of the trajectory. Compared to the simulation results in Table 6, the real platform naturally shows slightly higher error values due to disturbances and hardware imperfections that are not modelled. Nevertheless, the overall accuracy remains close to the simulation performance, demonstrating that the control approach transfers well from the model to the physical system.

Table 8 reports the Euclidean errors between the *MapBot* platform and the simulation, with and without orientation tracking. The metrics include RMSE, MAE, maximum error, 95th and 99th percentiles, and median. When orientation tracking is enabled, the position errors decrease significantly (RMSE drops from 0.0364 m to 0.0124 m), indicating improved localisation accuracy. However, orientation errors increase (RMSE from 0.0169 rad to 0.8058 rad), reflecting the additional rotational component in the error calculation. Without orientation tracking, position errors are larger, but orientation errors remain minimal, as the system does not account for rotational deviations. In general, the table demonstrates the trade-off between precise positional tracking and orientation-aware error metrics.

**Figure 22:**
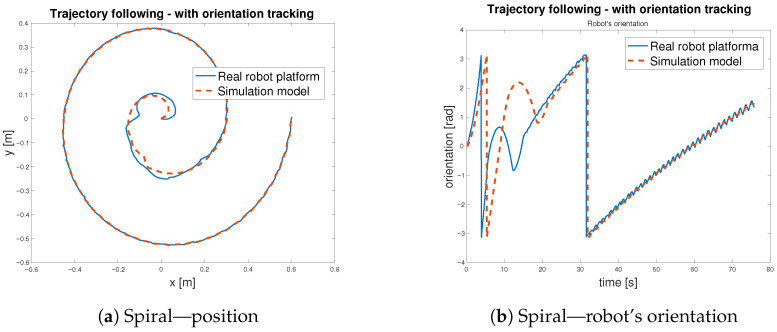
Trajectory following with orientation tracking—spiral.

**Figure 23:**
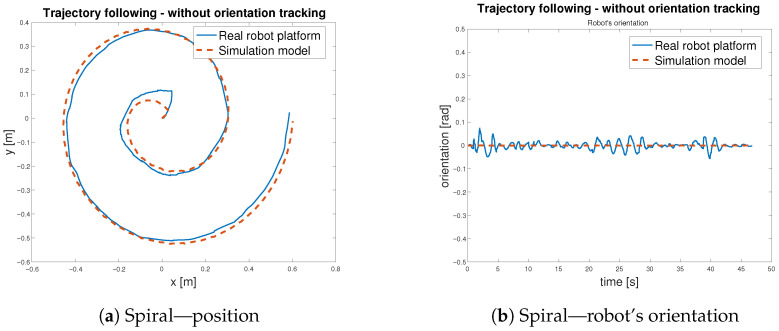
Trajectory following without orientation tracking—spiral.

The trajectory tracking is considered as an indirect validation of the digital twin of the *MapBot* platform. The implementation of the digital twin in MATLAB/Simulink considers the omniwheel chassis—the kinematic part of the model—as well as the estimated models of DC motors. Thus, the digital twin is created as a grey box model, combining the known parameters with the results of the experimental identification process. In addition, to match the behaviour of the real robot platform and its digital model, the simulation was set to use a fixed-step discrete solver aligned with the sample time of the real robot. The close convergence in Figure 22 and Figure 23 reflects the fidelity of the model under the robot’s sampled-data implementation rather than variable-step solver residuals.

### 6.3. SLAM

**Figure 24:**
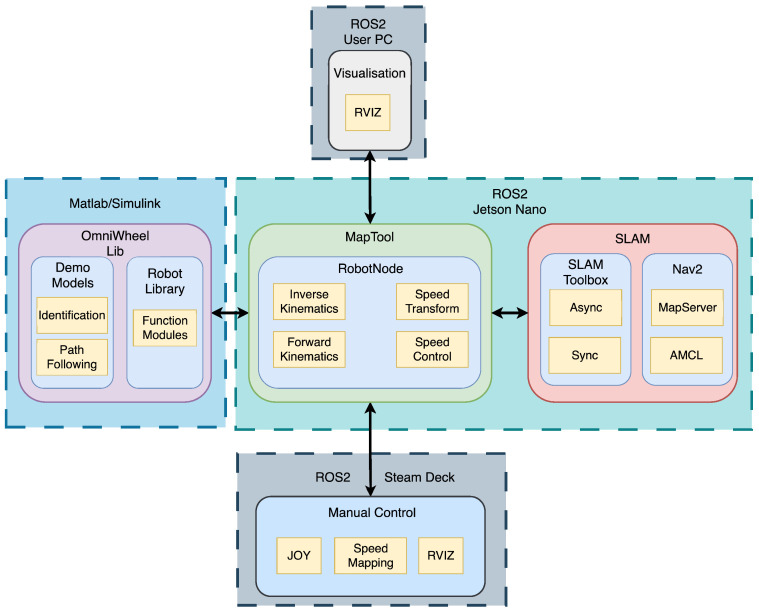
Software modules of *MapTool* application.

As the speed control and trajectory tracking algorithms of the mobile robot were successfully verified, the next step was to address the primary objective of the *MapBot* platform—Simultaneous Localisation and Mapping (SLAM). To achieve this goal, a dedicated application called *MapTool* was developed. The system integrates robot control, sensor data acquisition, and SLAM within a single framework (Figure 24). The application is built using the ROS framework and is executed within a Docker container based on the ROS 2 Humble image. This design choice ensures high portability, enabling the system to be easily migrated to different hardware platforms or operated remotely on external servers. It runs on an embedded Jetson Nano computer inside the robot, enabling on-board processing with low latency and avoiding the instability of wireless links. A SteamDeck gamepad controller provides teleoperation and live visualisation of the generated map. The system supports alternative game controllers compatible with the ROS2 Joy library, which allows flexible user interaction.

#### 6.3.1. Asynchronous Mapping and Localisation Using Pose Graph SLAM

**Figure 25:**
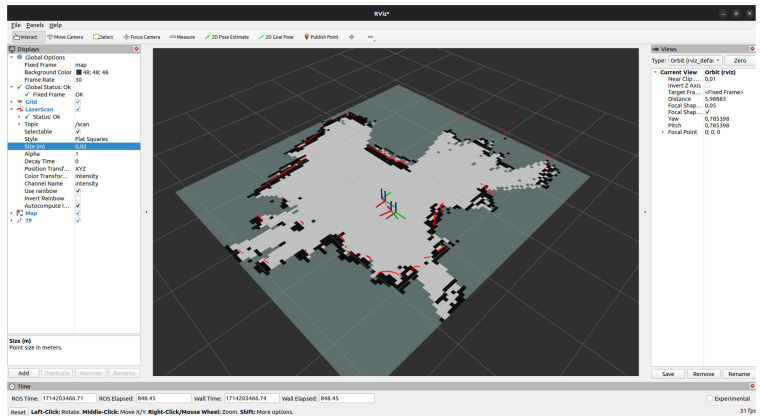
Map generated by asynchronous SLAM.

**Figure 26:**
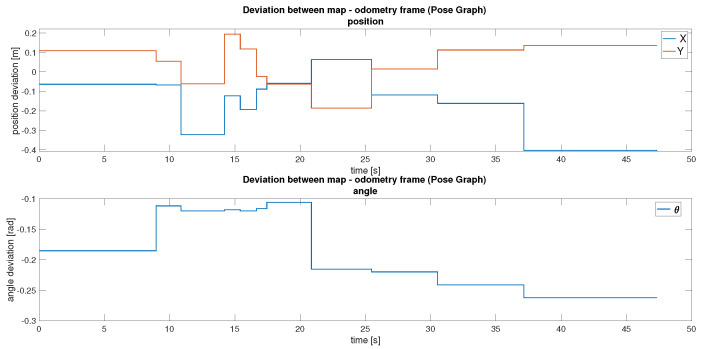
Difference between the map and odometry frames with pose graph.

The primary localisation and mapping approach in *MapTool* is asynchronous pose graph SLAM. This method continuously uses LiDAR scans and wheel odometry to build an occupancy grid map while estimating the robot’s pose. Asynchronous operation was chosen for its robustness to sensor dropouts and tolerance to imperfect message timing. An important feature of this approach is its ability to adapt the map over time. For example, if an obstacle is removed from the environment, it is gradually erased from the map. Localisation accuracy is achieved by correcting odometry drift through scan matching with map features, as illustrated in Figure 25. Although more computationally demanding than static-map localisation, this approach supports continuous updates in dynamic environments. The deviations between the map and odometry frames can be seen in Figure 26.

In practice, updates to the map frame occur at relatively long intervals. Although infrequent, these updates significantly improve localisation accuracy by realigning the robot’s position with the generated map. Efforts to increase the frequency of these corrections resulted in decreased map fidelity, indicating a trade-off between update responsiveness and the stability of the generated map.

Compared to traditional AMCL-based localisation, pose graph SLAM imposes higher computational demands. However, it offers the key advantage of supporting continuous map updates during operation, which is particularly beneficial in dynamic or partially unknown environments. This capability allows the system to adapt to changes in the environment, unlike AMCL, which relies on a static map.

#### 6.3.2. Localisation Using AMCL

**Figure 27:**
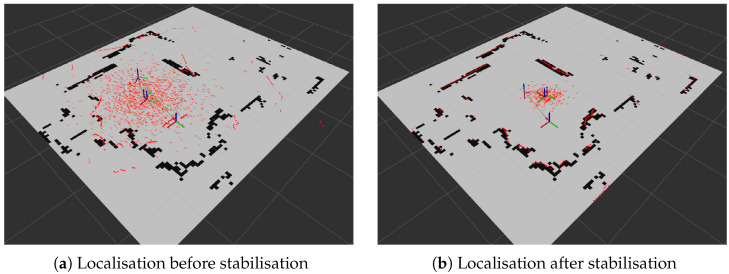
Localisation process using AMCL.

To evaluate an alternative approach, *MapTool* also supports Adaptive Monte Carlo Localisation (AMCL). In this case, a static map generated by asynchronous SLAM is used for localisation, and the robot’s position is estimated by particle filtering. As shown in Figure 27, the uncertainty in the position of the robot decreases as the robot moves and the sensor data align with the map features. AMCL is computationally efficient and simpler to configure but assumes a static environment and requires an initial pose estimate. This makes it less suitable for dynamic settings but attractive for applications where computational resources are limited.

The localisation results in Figure 27 depend strongly on the characteristics of the LIDAR sensor. In this study, an RPLidar (Slamtec) was used, which has a maximum range of approximately 6 to 12 m and operates with relatively low laser power compared to industrial grade sensors. These limitations primarily affect measurement quality: sensor performance degrades on low-reflectivity surfaces and exhibits increased noise at the far end of its range. Regardless of the sensor, the structure of the environment also plays a key role. Environments with rich and well-distributed features within the effective sensing range enable the AMCL particle filter to converge quickly, whereas sparse or large open areas provide fewer constraints for localisation, which can slow down stabilisation or increase uncertainty. Thus, while the results demonstrate successful localisation, they should be interpreted with awareness of both the limitations of the sensor and the environmental conditions under which it operates.

**Figure 28:**
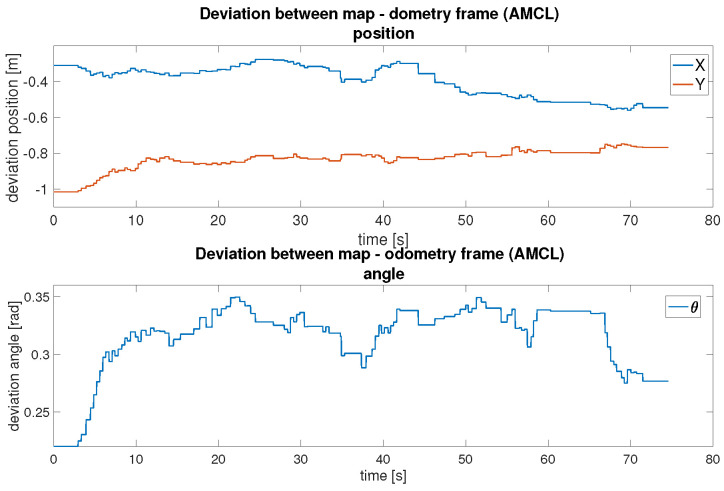
Difference between the map and odometry frames with AMCL.

Figure 28 presents the deviation map↔odom during AMCL localisation. Initially, the offset is high because of the pose uncertainty, but it quickly stabilises as the robot moves and the algorithm refines its estimate. Compared to pose graph SLAM, AMCL updates this transformation more frequently but relies on a static map. This trade-off simplifies computation but limits adaptability in dynamic environments.

In conclusion, both localisation methods were successfully implemented and evaluated. As shown in Table 9, the pose graph method demonstrates smaller maximum positional deviations in Y (0.194 m) and comparable deviations in X (0.407 m) compared to AMCL (X: 0.560 m, Y: 1.015 m). The pose graph method also shows a tighter orientation consistency (θ STD: 0.0639 rad vs. 0.0299 rad for AMCL), indicating more reliable rotational estimates across the trajectory. Asynchronous SLAM offers high adaptability and autonomous map updates, making it suitable for complex, changing environments, though at a higher computational cost. AMCL provides a more lightweight alternative for stable and known environments, requiring manual initialisation but offering simpler deployment, while exhibiting larger drift in positional measurements.

### 6.4. Comparison and Observations

In this case study, the primary focus was to design, implement, and evaluate a multi-layered CPS architecture in the context of the single *MapBot* platform. However, it is important to acknowledge that expanding the existing architecture to multiple robots is possible but not trivial. Before using multiple robots for SLAM tasks, a few constraints should be considered. First, when multiple robots share the same communication medium, interference can lead to packet collisions and transmission delays. To maintain reliable robot behaviour, the network must therefore be designed with sufficient capacity and robustness, tailored to the specific requirements of the application [47]. Second, data transmission requirements increase with the number of robots. To prevent raw sensor data from quickly exceeding bandwidth limits, efficient compression or submap-based sharing can be used to optimise data transmission [48]. Collaborative SLAM offers significant benefits by reducing data transmission requirements and computational overhead [49]. While the present work deliberately restricted itself to a single-robot setting to validate the CPS architecture, future extensions require addressing these scalability challenges in communication, bandwidth efficiency, and distributed coordination to enable robust multi-robot operation.

## 7. Conclusions

This paper presented the design, development, and evaluation of a modular mobile robot platform conceptualised as a Cyber–Physical System (CPS). The proposed architecture draws on established CPS and Distributed Control System (DCS) frameworks to organise the platform into five distinct but interrelated layers: component, control, coordination, supervisory, and management. This layered structure enables modularity, scalability, and clear functional separation between hardware and software elements, facilitating easier integration, extension, and future upgrades.

At the physical level, the robot integrates a range of sensing (stereo cameras, LiDAR, IMU, ToF sensors) and actuation systems (omnidirectional wheels), interconnected through a heterogeneous but structured communication framework using Ethernet, POE, I2C, and CSI interfaces. On the computational side, embedded systems such as the Jetson Nano and ESP32 microcontrollers manage perception, control, and data coordination across the layers. The ROS2 framework orchestrates these processes in a distributed fashion, supporting run-time visualisation, manual operation, and autonomous behaviour.

A significant part of this work focused on modelling and control-orientated contributions. A simulation model of the mobile robot was developed, followed by the experimental identification of DC motors. PID controllers and an FFC controller were designed for accurate trajectory tracking. The effectiveness of these controllers was indirectly validated by comparing the physical robot’s behaviour with its digital twin, demonstrating the fidelity of the simulation and the robustness of the control strategy.

As a practical application of the CPS framework, the platform was used to implement and compare real-time localisation and mapping techniques. Asynchronous pose graph SLAM enabled live map updates and adaptability in dynamic environments, though it required higher computational resources and was sensitive to odometry noise. In contrast, AMCL-based localisation provided a lightweight and stable solution for structured settings, relying on a prebuilt map and manual initialisation. Together, these experiments validated the robot’s ability to perform autonomous navigation tasks while illustrating the trade-offs between computational efficiency, responsiveness, and map quality.

In general, the integration of a CPS-inspired architecture with a real-world robotic platform provides a robust foundation for further research in autonomous systems, modular robotics, and digital twin applications. Future research may focus on advanced autonomy, multirobot coordination, remote monitoring via digital twins, or AI-enhanced decision-making embedded within the CPS framework.

## Figures and Tables

**Table 1:** Representative Architectures and Use-Cases of Cyber–Physical Systems.

Domain	Use-Case	Architecture Type	Functional Layers	Ref.
Industrial Automation	Autonomous production lines and factory-level control systems	ISA-95 Hierarchical Architecture	Device, Control, Supervisory, MES, Enterprise	[32]
Smart Grid	Renewable integration and distributed energymanagement	Decentralized Layered CPS	Sensor, Local Controller, Grid Controller, Cloud Interface	[33]
Scientific Instrumentation	Detector control system in ALICE at CERN	Multi-Layer CPS	Sensor, Frontend, SCADA/HMI, Database, Management	[5]
e-Health	Wearable health monitors with cloud diagnostics	IoT-Enabled CPS	Device, Gateway, Analytics, Clinical Interface	[34]
Smart Buildings	HVAC and lighting based on occupancy sensing	Human-Cyber Integrated Architecture	Sensing, Control, Interface, Optimization	[35]
Smart Agriculture	Precision irrigation and pest control via edge-cloud system	Edge-Cloud CPS	Sensors, Edge Devices, Cloud, Decision Logic	[36]
Smart Mobility	Vehicle coordination with V2X and onboard AI	Distributed ReactiveCPS	Sensors, Vehicle Control, Communication, Cloud	[37]

**Table 2:** Physical quantities used in mobile robot platform.

Symbol	Description
$v_{x}$	Lateral velocity in X axis
$v_{y}$	Lateral velocity in Y axis
$\omega_{z}$	Angular velocity around Z axis
$\omega_{FL}$	Angular velocity of front-center wheel
$\omega_{FR}$	Angular velocity of front-right wheel
$\omega_{RL}$	Angular velocity of rear-center wheel
$\omega_{RR}$	Angular velocity of rear-right wheel

**Table 3:** Mobile robot platform parameters.

Symbol	Description
*r*	Wheel radius
$L_{y}$	Distance between wheels along Y axis
$L_{x}$	Distance between wheels along X axis
$\left[0_{G},X_{G},Y_{G}\right]$	Global coordinate system
$\left[LSS,X_{R},Y_{R}\right]$	Local coordinate system

**Table 4:** Errors of ARMAX model identification for DC motor angular speed [rad/s].

RMSE	MAE	Max	p95	p99	Median
0.24686	0.17247	3.4968	0.32553	0.55001	0.15125

**Table 5:** Validation of DC motor control algorithm (closed-loop) [rad/s].

RMSE	MAE	Max	p95	p99	Median
0.078773	0.063882	0.22337	0.1542	0.17903	0.054748

**Table 6:** Euclidean errors between the reference trajectory and the simulation [m].

Case	RMSE	MAE	Max	p95	p99	Median
Sine wave	0.0023439	0.0018977	0.0051059	0.0042061	0.0046162	0.0016826
Square	0.0079314	0.0064505	0.022282	0.01662	0.019944	0.0051852

**Table 7:** Euclidean errors between the reference trajectory and the *MapBot* platform.

Case	RMSE	MAE	Max	p95	p99	Median
Position [m]	0.028164	0.027121	0.058838	0.040388	0.050839	0.026027
Orientation [rad]	0.016946	0.012048	0.074502	0.036537	0.050123	0.0079494

**Table 8:** Euclidean errors between the *MapBot* platform and the simulation.

Case	RMSE	MAE	Max	p95	p99	Median
**With orientation tracking**						
Position [m]	0.012377	0.0091677	0.041236	0.028306	0.038394	0.0064026
Orientation [rad]	0.80579	0.39621	2.9347	2.2492	2.8797	0.092072
**Without orientation tracking**						
Position [m]	0.036404	0.034774	0.075434	0.054943	0.061782	0.034284
Orientation [rad]	0.016948	0.012051	0.074225	0.036617	0.049943	0.0079975

**Table 9:** Drift measurement statistics.

Parameter	AbsMax	AbsMin	STD	MaxAbsDiff
**Pose graph method**				
X	0.40723	0.0588	0.14588	0.25478
Y	0.19379	0.014701	0.11055	0.25538
θ	0.26238	0.10584	0.063866	0.10962
**AMCL**				
X	0.56019	0.27671	0.088628	0.056593
Y	1.0151	0.74711	0.05883	0.040554
θ	0.34966	0.22016	0.029948	0.016952

## Data Availability

Data are contained within the article.

## Abbreviations

The following abbreviations are used in this manuscript:

ALFRED	ALICE Low-Level Front End Device
ALICE	A Large Ion Collider Experiment
ALICE-DCS	ALICE Detector Control System
AMCL	Adaptive Monte Carlo Localisation
CERN	Conseil Européen pour la Recherce Nucléaire (European Organisation for Nuclear Research)
CPS	Cyber–Physical System
CMCT&II	Center of Modern Control Techniques and Industrial Informatics
DCS	Distributed Control System
FRED	Front End Device
ES	Embedded System
HMI	Human–Machine Interface
LHC	Large Hadron Collider
NCS	Networked Control System
SCADA	Supervisory Control and Data Acquisition
SLAM	Simultaneous Localisation and Mapping
